# Age- and sex-specific differences in repetitive sprinting in 9-14-year-olds living in Turkey

**DOI:** 10.1186/s12889-025-21703-w

**Published:** 2025-02-11

**Authors:** İbrahim Can, Bilgin Ataş, Bouwien C. M. Smits-Engelsman

**Affiliations:** 1 Department of Coaching, Faculty of Sport Sciences, Iğdır University, Iğdır, Turkey; 2 Secondary School of Mustafa Yassuboğa, Doğu Beyazıd, Ağrı, Turkey; 3 Physical Activity, Sport and Recreation, (PhASRec, focus area, Faculty Health Sciences, North-West University, Potchefstroom, South Africa

**Keywords:** Children, Physical activity, Anaerobic power, Repeated sprint, Age, Sex

## Abstract

**Background:**

Since children’s daily activities are mostly anaerobic, it is important to assess anaerobic performance as part of the physical assessment. Therefore, running speed, power, and heart rate during repetitive sprints were investigated.

**Method:**

A total of 118 children participated (mean age:11.5 range 9-14y; 48% boys, 52% girls) and performed the children’s repetitive and intermittent sprinting performance (CRISP) test, which consists of six sprint runs performed at maximal speed over 30-meter with short recovery periods (10 s) between each run. GLM Repeated Measures were used to examine the effect of runs (within-subject factor) and age and sex (between-subject factor) and as well as possible interactions for running time, power, and heart rate.

**Results:**

Running times increased significantly across runs (*p* < 0.001, large effect size), showing a non-linear slowdown (*p* = 0.013). Also, a main effect of age, [*p* < 0.033] and sex [*p* < 0.011, medium effect size] emerged. However, interaction effects showed that girls fatigued more in the second half of the test, which led to larger differences with the boys in the later runs of the CRISP. Moreover, the interaction effect of age-by-run-by-sex was found. Eleven-year-old children had the longest running times. At the same time, young children, predominantly boys, showed less fatigue, as their last runs were comparable in time to the first ones. Analysis of the power showed a main effect of age, indicating more power in older children [*p* < 0.001, large effect size], especially after 11 years. No differences between sexes were found for power. Heart rate increased significantly during the repetitive sprinting [*p* < 0.001, large effect size]. No differences for age or sex were found for mean or peak heart rate.

**Conclusion:**

The CRISP test is sensitive to running fatigue [large effect size] and distinguishes between the performance characteristics of children according to age and sex. Running speed and power go up between 12 and 14 years. Girls run slower but generate comparable power over the runs. Yet they fatigue more in the second half of the test than boys.

## Background

Active games are one of the best ways of engaging children in physical activity. Children who play active games easily push themselves to their limits for a few seconds [1]. Children typically favor intermittent, brief exercises over continuous activity, which aligns with their natural activity patterns of short, intense efforts followed by recovery [2, 3]. For instance, Bailey et al. [4] observed children aged 6–10 and found a median duration of three seconds for intense activities, while 95% of intense activities lasted less than 15 s, highlighting the predominance of anaerobic effort in these activities. Thus, repeated short-term high-intensity activities are more characteristic of the spontaneous physical activity of children and are performed regularly in daily tasks, games or sporting events [3, 5].

Short bursts of physical activity utilize muscle strength, power, and muscular endurance and mostly engage the anaerobic energy system [6]. Anaerobic performance, the ability to sustain high-intensity activity over short periods [7, 8], plays a crucial role in children’s overall physical health and may help prevent future cardiovascular, metabolic, and mental health issues [9].

Assessing anaerobic performance is vital for understanding children’s fitness levels. Common field tests, such as vertical jumps, long jumps, and sprints, can provide useful information [1, 6, 10, 11]. Sprinting can be defined as running at high speed for a short time [12]. Repeated sprinting is commonly used to examine fatigue mechanisms related to high-intensity intermittent sports and recreational activities and is defined as sprints that are short (≤ 10 s) in duration with short recovery periods (≤ 60 s) between sprints [13, 14, 15].

Sprinting, particularly repeated sprints with short recovery, is a key component of anaerobic fitness. Two very similar tests for assessing repeated sprinting in children are the Pediatric Run-based Anaerobic Sprint Test (RAST) and the Children’s Repetitive and Intermittent Sprinting Performance (CRISP) test. The RAST, consisting of six 15-meter sprints with 10-second recovery, has been validated for children aged 6–18 years [15]. This test consists of six repetitive sprints of 15 m interspersed with short recovery periods (10 s). In the mentioned study, no significant difference was obtained in terms of sex in peak power (PP) and mean power (MP) but a strong relationship was obtained with age.

In an earlier study, Smits-Engelsman and Bonney [16] found that the average running time for the 15 m RAST was less than 4 s for children and times were not different for boys and girls [17]. Further, they noticed no significant decrease in running speed. Therefore the running distance was increased to 30-meter in the CRISP test. The CRISP test, which was used in this study, was designed to measure both anaerobic performance and fatigue, involves six 30-meter sprints with 10-second rest intervals (see methods). This test has shown promise in evaluating children’s anaerobic fitness and fatigue in a younger age group [16].

Age and sex are key factors influencing anaerobic performance. Age is the common reference in growth and performance research. The anaerobic fitness of children is lower than that of adults, and therefore their ability to perform anaerobic type activities such as throwing, jumping, swimming, and sprinting is significantly lower than adults and adolescents [18, 19].

Sex is also an important factor [20, 21]. Several authors have pointed out that increased anaerobic power is mainly related to the development of (lean) muscle mass [22, 23, 24]. Although the natural physiological adaptations in the structure of men and women are similar in many points, there are anatomical and physiological differences [25]. Skeletal muscle constitutes approximately 50% of body mass in men and 40% in women [26]. The differences in muscle mass, and leg length likely contribute to variations in running performance and fatigue resistance, with boys generally showing higher sprint performance and greater endurance [27].

Given the limited number of studies, there is a clear need to further explore age and sex differences in repetitive sprinting as a measure of anaerobic performance and of fatigue (decay over runs). On most fitness measures boys outperform girls, and older children the younger ones, but no data are available using the CRISP on possible differences in running time, fatigue or heart rate. Therefore, this study aims to investigate the impact of age and sex on running time, power, fatigue, and heart rate during the CRISP test. It is hypothesized that running time will increase over runs, that boys will perform better than girls in terms of running time and fatigue resistance, and that younger children will produce lower power than older children due to differences in muscle mass [28, 29]. No difference in heart rate is expected according to age and sex [30].

## Materials and methods

### Study design and setting

This cross-sectional study included participants of different ages and sex who attend regular primary and secondary school in a village in Iğdır province, located in the easternmost part of Turkey. Although sporting areas in the village and the school are not adequate, this study occurred in a primarily agricultural community where children are exposed to challenging living conditions (gardening, husbandry, etc.) from an early age.

### Participants and calculation of sample size

All children in grades 4-8th (between the ages of 9 and 14 years) who received signed written informed consent from their parents, signed assent, and did not have a history of disability that could negatively affect running performance were eligible to be included in this study (*n* = 118). The study sample size was calculated through a power analysis for a mixed ANOVA that showed that a total sample size of 108 was needed for a medium effect size (f = 0.20), at a power of 95%, while alpha is set at 0.05. The G-power analysis software version 3.1 was used for the sample size calculation [31].

### Procedures

All participants were tested during their free time in the spring period of 2023 and the measurements were completed within three weeks. The measurements were taken between 11:30 and 13:00, outside on a non-slip clay playground in a public school. The study received ethical approval from the Scientific Research and Publication Ethic Committee of the University of Iğdır (SRPEC approval no 20/12-5-2022) and was performed in accordance with the Declaration of Helsinki. Permission was obtained from the principal of the school and designated education authorities in the Iğdır Province of Turkey.

### Measurements

#### Demographic and anthropometric characteristics

Demographic data including age, sex and grade were collected. Height was measured using a steel tape measure (Fisko Tri-Lok, UK) in the anatomical position and without shoes, while body weight was measured using an electronic scale (Medishop BY-810, Uruguay) without shoes and in light clothing. Body mass index (BMI) was calculated using the formula based on body weight and height (kg/m²).

#### The children’s repetitive and intermittent sprinting performance (CRISP) test

Children’s repetitive and intermittent sprinting performance (CRISP) test [16] was used to measure anaerobic fitness and fatigue. The test consists of six sprint runs performed at maximal speed over 30-meter with short recovery periods (10 s) between each run. Participants were instructed to run from one line to the other as fast as possible and not to slow down before crossing the finish line. Sprint times were measured using an electronic stopwatch (TFA Dostmann, Germany). Participants were given a one-minute standardized warm-up protocol before implementation of the test protocol and allowed a practice trial. Verbal encouragement was given to all participants during the test.

For the comparison between age and sex, outcomes based on the sprint times were calculated. Mean power (MP) was used as a measure of anaerobic capacity and calculated using the sprint time of the six runs and the weight of each participant. Power was computed using a formula [power = (body mass×distance^2^)/sprint time^3^] [32]. Peak power (W) was the highest power output of all six sprints. Greater MP indicates the ability to maintain power output over time and translates into better maintenance of anaerobic performance. The participants’ heart rates were determined using an electronic watch (Haylou Solar Smart Watch LS05 Global, Dongguan Liesheng Electronic, China) at rest and after each run. Participants sat down on a chair when they arrived for one minute and their resting heart rate values were taken. After completing each 30-meter sprint run, participants’ heart rates were recorded by an assistant during the 10 s resting period. Estimated maximum heart rate was computed using the formula: Estimated maximum heart rate (EMHR_max_) = 206 - (0.88 x age) [33]. Also, the percentage of the estimated maximum HR reached after the runs was calculated to estimate training intensity.

### Statistical analysis

Statistical analyses were performed with SPSS 29.0 (SPSS Inc., IBM Company, Armonk, NY). The normal distribution of the variables was confirmed using visual check of histograms, Q–Q, box plots and Kolmogorov-Smirnov test. Demographic data (age, sex, height, weight, BMI) were used to describe the sample. Independent t-test and ANOVA were used for comparison between the sex and age, respectively for demographic data; Chi-squared to compare the frequency of sex between age groups. GLM Repeated Measures were used to examine the effect of runs (within-subject 6 levels) with age group and sex (between-subject factors) and their possible interactions for Time, Power, and the same analysis was performed for Heart rate (within-subject 7 levels). Post hoc tests were corrected for multiple testing. Effect sizes were calculated using partial eta squared (η²), with the following interpretations: small η² = 0.01, medium η² = 0.04, and large η² > 0.14 [34].

## Results

Demographic values of 118 students regarding their distribution according to sex and age are given in Table 1. The mean age for the boys was 11.61 (SD 1.73) and 11.31 years (SD 1.60) for the girls (p 0.34). Moreover, no significant differences in height, weight, and BMI were found between boys and girls. Also, the frequency of boys and girls was not different between the age groups (p 0.82).

**Table 1:** Demographic and anthropometric values of students according to age and sex

	Group	Number	Percent	Weight (kg) Mean (± sd)	Height (cm) Mean (± sd)	BMI Mean (± sd)
Sex	Boys	64	54.2	38.6 ± 10.7	146.9 ± 11.6	17.84 ± 3.96
	Girls	54	45.8	40.1 ± 9.5	145.4 ± 9.1	18.87 ± 4.05
	Total	118	100	39.3± 3.9	146.1 ± 10.5	18.31 ± 4.02
Age	9	19	16.1	30.1±4.2	137.6±7.1	16.03 ± 2.60
	10	20	16.9	32.9±8.3	138.3±6.2	17.24 ± 4.11
	11	20	16.9	38.0±7.8	142.3±6.3	18.97 ± 4.73
	12	21	17.8	40.5±7.7	147.6±7.3	18.81 ± 4.42
	13	21	17.8	44.3±7.1	152.4±7.3	19.18 ± 3.53
	14	17	14.4	52.8±8.7	161.8±7.2	19.62 ± 3.54

### Main effects and interactions on running time

Statistics are summarized in Table 2 and means in Table 3.

**Table 2:** Results of the repeated measures ANOVA (main effect and interactions) for the running time of the CRISP test

Variable	df	F-value	*p*-value	Partial Eta Squared
Age	5,106	2.54	= 0.033	0.107
Sex	1,106	6.63	= 0.011	0.059
Age * Sex	5,106	1.45	0.214	0.064
Runs	5,102	24.23	< 0.001	0.543
Runs * Age	25,530	1.13	= 0.307	0.050
Runs * Sex	5,102	3.85	0.003	0.159
Runs * Age * Sex	25,530	1.80	= 0.011	0.078

Abbreviations: df - degrees of freedom

**Table 3:** Means and 95% confidence intervals for time (s) per run. Values as grand mean, and for sex and age groups

Run	Mean	Lower CI	Upper CI	Mean	Lower CI	Upper CI	Mean	Lower CI	Upper CI
	Grand mean for Time			Boys Time			Girls Time		
1	6.08	5.99	6.17	6.04	5.92	6.16	6.12	5.99	6.25
2	6.21	6.10	6.32	6.13	5.98	6.27	6.30	6.14	6.46
3	6.36	6.26	6.47	6.29	6.15	6.43	6.44	6.28	6.59
4	6.36	6.26	6.46	6.23	6.10	6.37	6.49	6.34	6.64
5	6.50	6.39	6.62	6.28	6.13	6.43	6.73	6.56	6.90
6	6.54	6.42	6.66	6.37	6.21	6.53	6.70	6.53	6.88
	9 years Time			10 years Time			11 years Time		
1	6.21	5.99	6.43	6.14	5.92	6.35	6.31	6.10	6.52
2	6.18	5.92	6.44	6.41	6.16	6.67	6.47	6.21	6.72
3	6.39	6.13	6.64	6.52	6.27	6.77	6.67	6.42	6.92
4	6.29	6.05	6.54	6.46	6.23	6.70	6.60	6.37	6.84
5	6.32	6.05	6.60	6.68	6.41	6.95	6.86	6.59	7.13
6	6.36	6.06	6.65	6.70	6.42	6.98	6.91	6.63	7.19
	12 years Time			13 years Time			14 years Time		
1	5.92	5.71	6.13	5.99	5.78	6.20	5.91	5.66	6.17
2	6.08	5.83	6.34	5.97	5.72	6.22	6.15	5.85	6.46
3	6.25	6.01	6.49	6.22	5.97	6.46	6.12	5.83	6.42
4	6.33	6.10	6.56	6.29	6.06	6.52	6.18	5.91	6.46
5	6.48	6.22	6.74	6.41	6.15	6.67	6.27	5.95	6.58
6	6.45	6.17	6.73	6.49	6.21	6.76	6.32	5.98	6.65

**Fig. 1:**
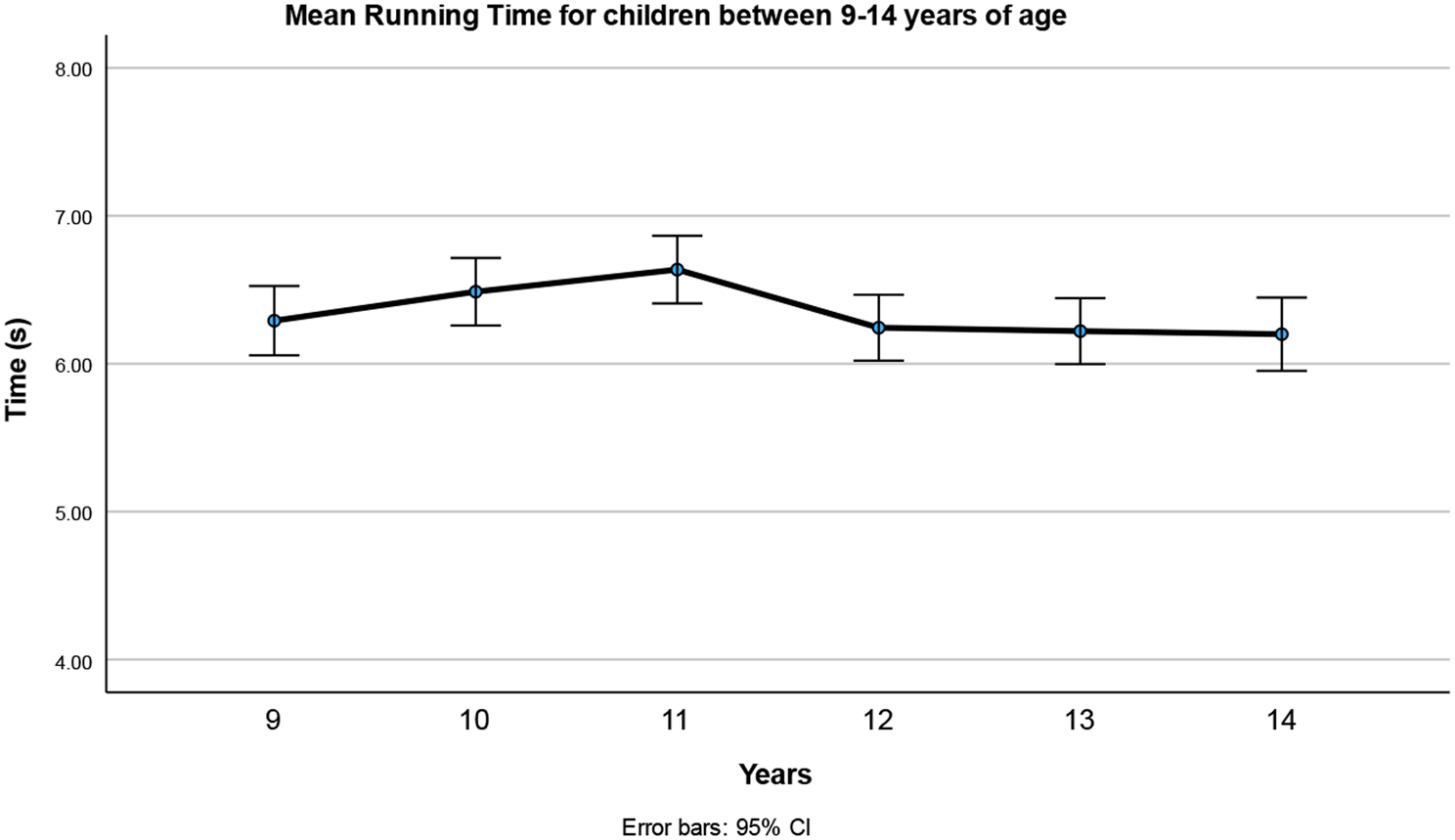
Time to complete the six 30-m sprints of the Children’s Repetitive and Intermittent Sprinting Performance (CRISP) test in 9–14-year-old children (data are means ± 95%CI. Time at age 11 was different from the 9,12, 13, and 14 years old; *p* = 0.033, p-0.014, *p* = 0.0, *p* = 0.007, respectively)

Repeated measures ANOVA showed a main effect of age. However, this effect was not linear because the youngest age group was faster than the 11-year-olds (*p* = 0.032) (Fig. 1). Also, 12, 13, and 14-year-olds were faster than the 11-year-olds (*p* = 0.014, *p* = 0.01 *p* = 0.007, respectively), but not from each other.

**Fig. 2:**
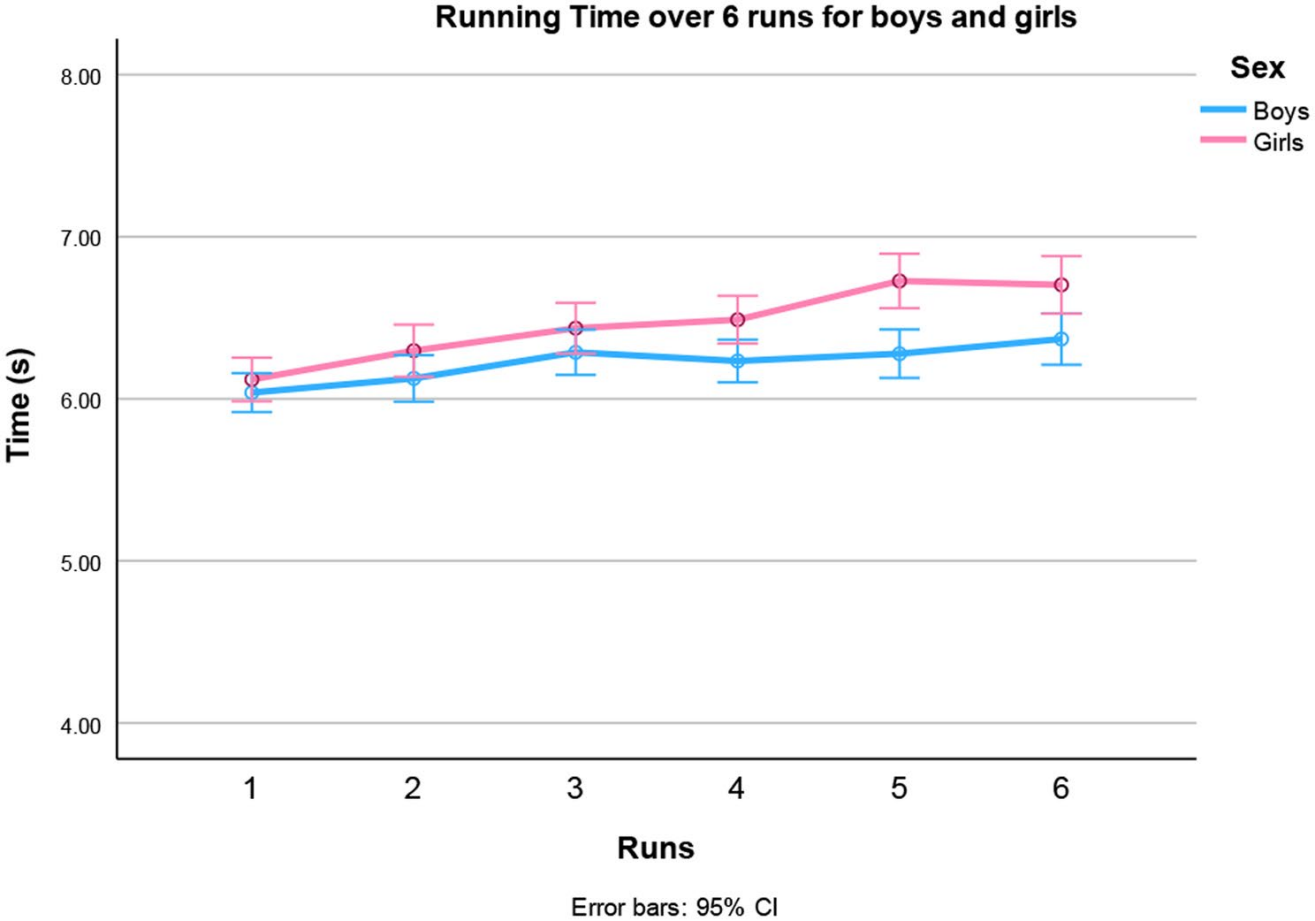
Time to complete the six 30-m sprints of the Children’s Repetitive and Intermittent Sprinting Performance (CRISP) test for boys and girls (data are means ± 95%CI. *p* < 0.011 for sex). Girls had longer running times than boys, especially in the later runs, while boys finished in more comparable times in runs 3–6

The main effect of sex was also significant indicating poorer performance among the girls in running time (Fig. 2). On average, they were 4.6% slower than boys.

**Fig. 3:**
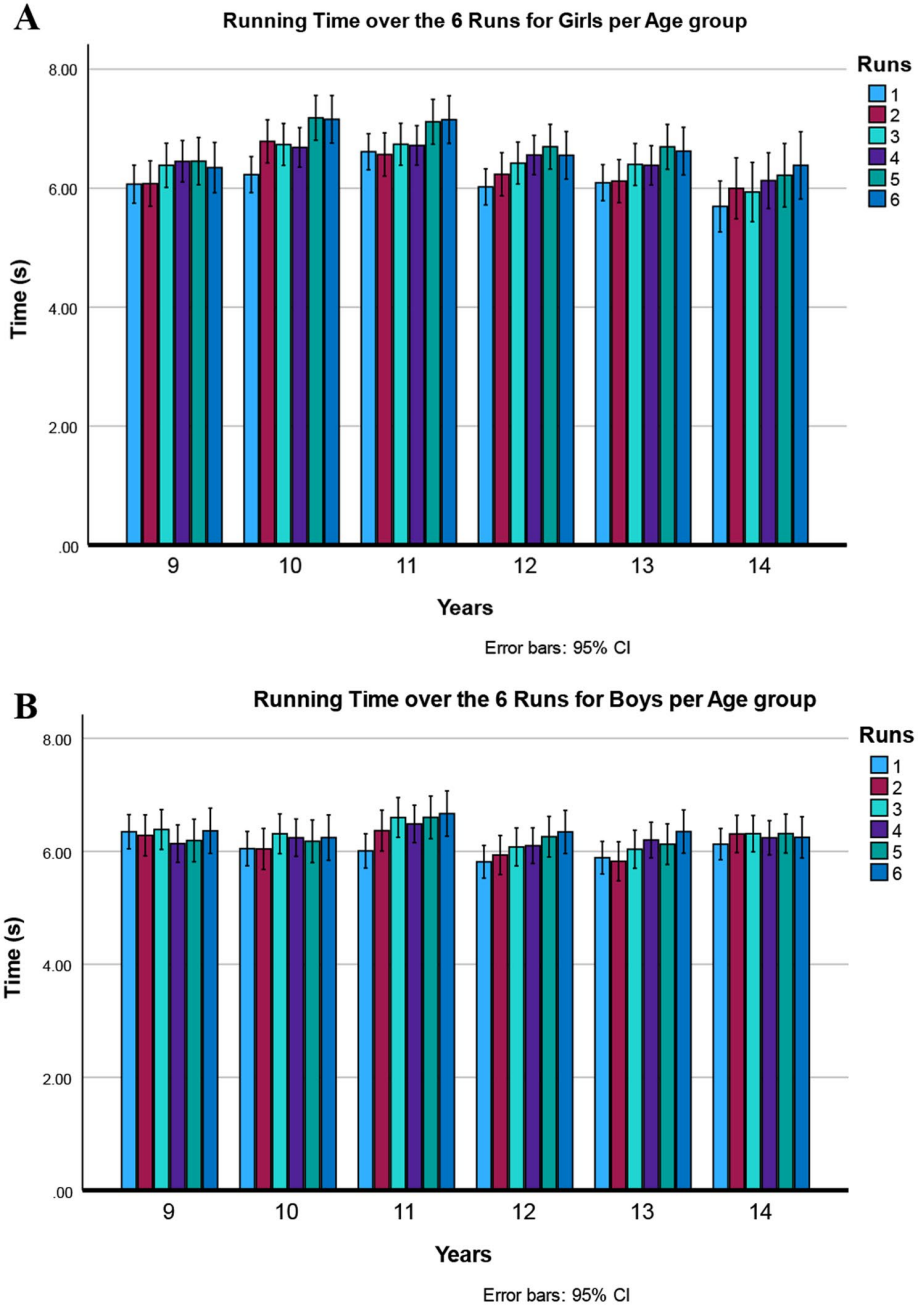
Depicts the three-way interaction of run by age by sex (data are means ± 95%CI.; *p* = 0.011). **A** Top Time to complete the six 30-m sprints of the Children’s Repetitive and Intermittent Sprinting Performance (CRISP) test in 9–14-year-old girls. **B** Bottom Time to complete the six 30-m sprints of the Children’s Repetitive and Intermittent Sprinting Performance (CRISP) test in 9-14-year-old boys (*P* < 0.033). Girls showed a gradual increase in running time in all age groups. Boys showed less increase overall and no increase in time over the 6 runs in 9- and 14-year-olds

A large main effect of runs was found on time. However, a polynomial higher-order effect was significant [F (1,106) = 6.39, *p* = 0.013], indicating a non-linear slowdown over the runs. (See Fig. 2). A two-way interaction of run by sex was found indicating that the differences are larger between boys and girls in later runs. Moreover, a three-way interaction of run by age by sex was also significant. This interaction was caused by the lack of sex differences between the first runs (Fig. 2) and because differences in running times between the runs were larger in girls (Fig. 3A) than boys (Fig. 3B). The youngest and oldest boys did not significantly change their running times over the runs (9 years p 0.72, 14 years p 0.08). Girls, however, had a more comparable pattern of gradually slowing down over the runs in the different age groups. (See Fig. 3A).

### Main effects and interactions on power

**Fig. 4:**
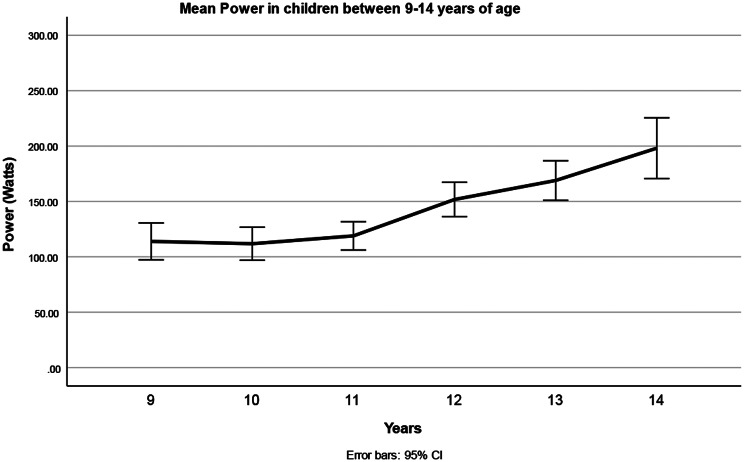
Power generated to complete the six 30-m sprints of the Children’s Repetitive and Intermittent Sprinting Performance (CRISP) test in 9–14-year-old children (data are means ± 95%CI, Main effect Age *p* < 0.001). The total power over the 6 runs increased after the age of 11

Statistics are summarized in Table 4 and means in Table 5. Repeated measures ANOVA showed a main effect of age group, indicating more power in older children. The main effect of sex was not significant. The power increased with age, especially after 11 years (See Fig. 4).

**Table 4:** Results of the repeated measures ANOVA (main effect and interactions) for the power of the CRISP test

Variable	df	F-value	*p*-value	Partial Eta Squared
Age	5,106	14.09	< 0.001	0.399
Sex	1,106	1.04	= 0.311	0.010
Age * Sex	5,106	0.23	0.949	0.011
Run	5,102	27.14	< 0.001	0.571
Run * Age	25,530	1.63	0.029	0.071
Run * Sex	5,102	2.61	0.029	0.113
Run * Age * Sex	25,530	1.64	0.027	0.072

Abbreviations: df - degrees of freedom;

**Table 5:** Means and 95% confidence intervals (CI) for the power (Watts). Values as grand mean, and for sex and age groups

Run	Mean	Lower CI	Upper CI	Mean	Lower CI	Upper CI	Mean	Lower CI	Upper CI
		Grand mean Power		Boys Power			Girls Power		
1	164	155	174	164	151	176	165	151	179
2	154	146	162	157	146	167	152	140	163
3	144	136	151	145	135	155	143	132	154
4	142	135	149	147	138	157	137	126	147
5	135	127	143	143	133	153	127	116	138
6	134	126	142	140	129	151	128	115	140
	9 years Power			10 years Power			11 years Power		
1	119	96	142	133	111	156	141	118	163
2	121	102	140	116	98	135	131	113	150
3	112	93	130	111	93	128	117	99	134
4	112	95	129	114	97	131	121	105	138
5	115	97	134	106	88	123	109	91	127
6	115	94	135	104	84	123	107	88	127
	12 years Power			13 years Power			14 years Power		
1	178	157	200	190	168	212	225	198	252
2	166	148	184	194	176	212	196	173	218
3	153	136	170	170	152	187	202	181	223
4	145	128	161	168	151	184	194	174	214
5	138	120	155	155	137	172	187	166	209
6	140	121	160	150	130	169	187	164	211

**Fig. 5:**
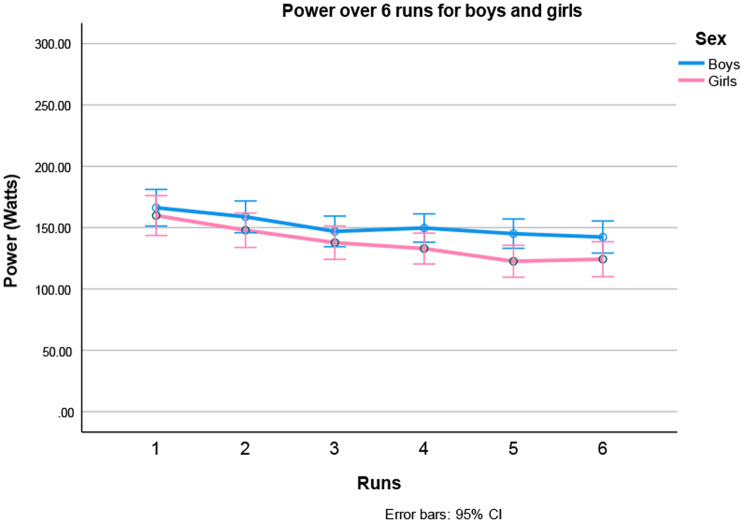
Power generated to complete the six 30-m sprints of the Children’s Repetitive and Intermittent Sprinting Performance (CRISP) test in boys and girls (data are means ± 95%CI). With repetition of the runs, the power (Watts) goes down (Main effect Runs: *p* < 0.001)

**Fig. 6:**
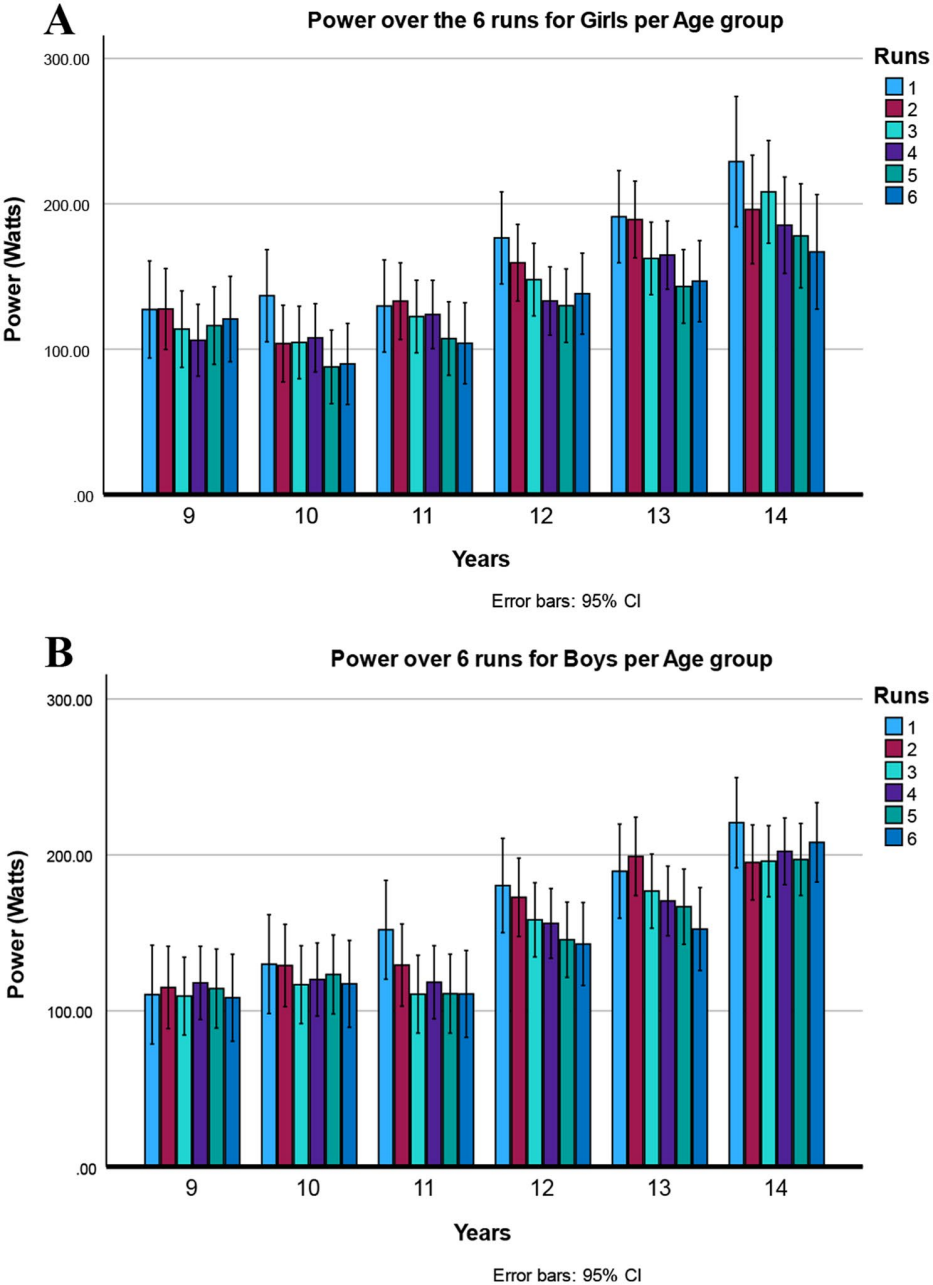
Depicts the three-way interaction age by sex by run (data are means ± 95%CI; *p* = 0.027). **A** Top Power generated to complete the six 30-m sprints of the Children’s Repetitive and Intermittent Sprinting Performance (CRISP) test in girls. **B** Bottom Power generated to complete the six 30-m sprints of the Children’s Repetitive and Intermittent Sprinting Performance (CRISP) test in girls. The spread in the values over the repetitions of the runs is less in young children (9-year-old girls and 9 and 10-year-old boys), they generate more comparable power over the 6 runs, indicating less fatigue

Also, a large main effect of run was found, contrast analysis showed that this effect was quadratic [F (1,106) = 8.274, *p* = 0.005], which was caused by a larger loss of power in the first runs. Girls lost more power than boys in the last runs, confirmed by the sex-by-run interaction (See Fig. 5). Moreover, the three-way interaction (age by sex by run) was also significant. For a more detailed description of this interaction, see Fig. 6.

### Main effects and interactions on heart rate

**Fig. 7:**
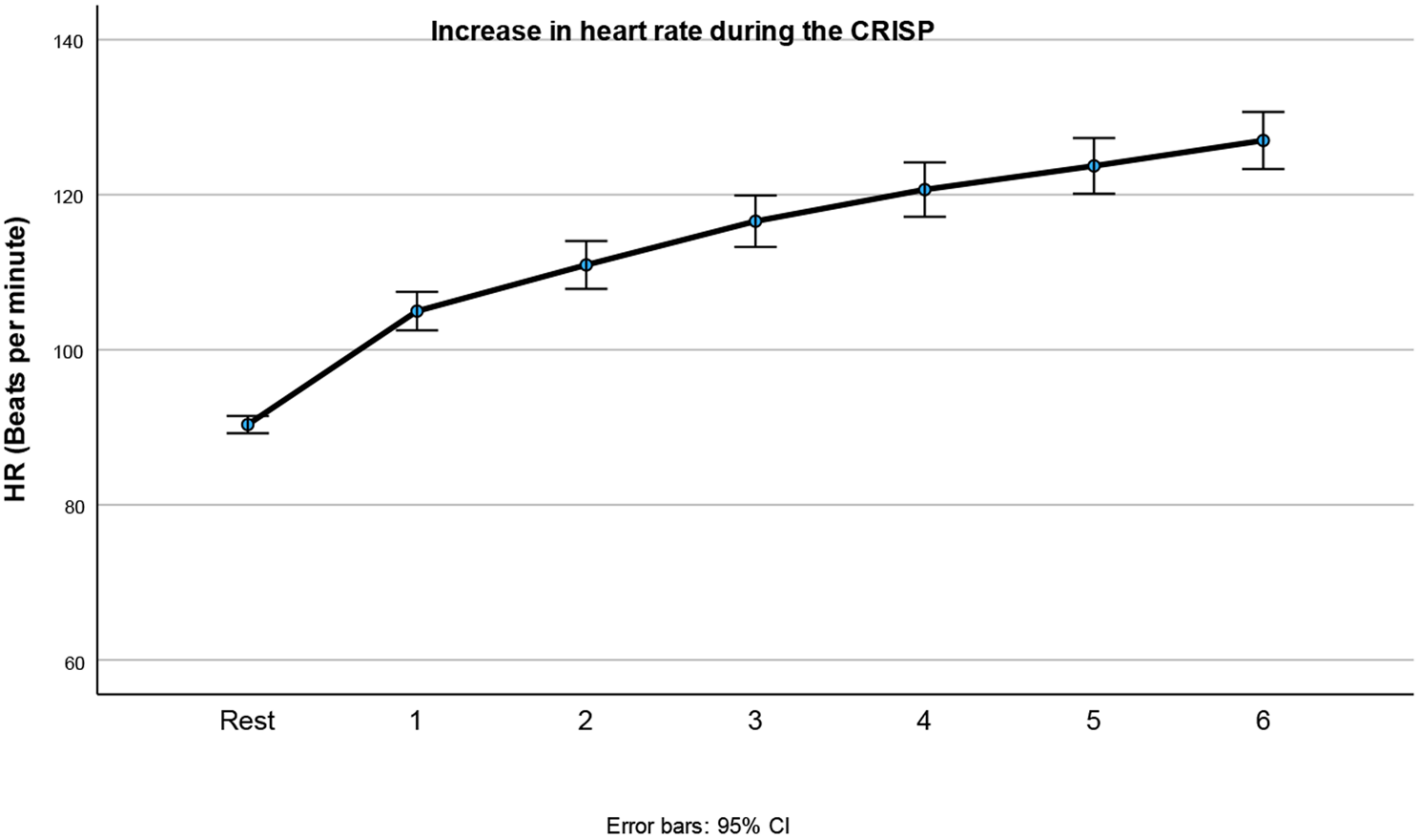
Depicts the increase in heart rate (HR) over the 6 runs. Main effect of Runs: *p* < 0.001. First measurement is rest HR

No main effects of age or sex were found for HR. Heart rate increased significantly during the repetitive sprinting [F (6,101) = 57.31; *p* < 0.001, eta 0.77; Table 6; Fig. 7] and this increase was comparable for the different age groups and sex (no interactions). Most children reached between 60 and 76% of their estimated maximum heart rate immediately after their sprint (Table 7).

**Table 6:** Mean and 95% confidence interval (CI)) of the heart rate per run

Heart rate (bpm)	Mean	Lower Bound CI	Upper Bound CI
Before start	91	89	92
After run 1	105	102	107
After run 2	111	108	114
After run 3	116	113	120
After run 4	121	117	124
After run 5	124	120	128
After run 6	127	123	131

**Table 7:** Intensity level based on estimated maximum heart rate (EMHR) during the 6 runs of the CRISP

% of EMHR	*N*	%
40–59%	33	28.0%
60–76%	73	61.9%
> 77%	12	10.2%
Total	118	100.0%

## Discussion

Sprinting, particularly repeated sprints with short recovery, is a key component of anaerobic fitness and is commonly used to evaluate fatigue mechanisms. Resistance to fatigue and the ability of muscles to recover during high-intensity intermittent exercise are important attributes of neuromuscular function and have been linked to both biological maturation [28] and sex differences [35]. In this study, we explored how age and sex affect running time, power, and heart rate during the CRISP test in children aged 9–14 years. Running fatigability was explored by calculating the increase in running time across six repetitions. Results confirmed that repeated sprints with short recovery intervals led to a 6.8% increase in time needed to cover the run of 30 m. The youngest children demonstrated less decay in running time, maintaining almost the same speed throughout. Heart rate increased from 90 bpm to 126 bpm by the last run, but no significant differences were observed between age groups or sexes in heart rate responses.

### Running time

Contrary to our expectations, running speed did not improve linearly with age. The 10-year-old children showed comparable performance to the 11-year-olds. The 9-year-olds ran faster than the 11-year-olds, not due to starting speed but because they maintained a more consistent pace across the six runs, whereas older children (11–13 years), particularly girls, slowed down. This resulted in more dispersed values between age groups in girls than in boys (see Fig. 2). Boys at the lowest and highest ages participating in the study showed the least change in running time. In contrast, girls exhibited a gradual slowdown across runs, with a more consistent pattern of deceleration across age groups.

### Power

Power improved significantly after age 11. As children grow older, body mass increases, which is used in the formula to calculate power. Boys experience a more significant increase in lean body mass during puberty, which is associated with improved power [36]. However, it is not known how much of the gained weight in the current age groups was lean body mass versus fat. Sex differences were smaller in children under 10 years, with larger differences emerging after puberty, aligning with findings that boys begin to outperform girls around age 12 [20, 37]. Muscle mass and hormonal changes likely explain the differences in performance, with males benefiting from greater muscle mass and cardiovascular capacity. Sex differentiation in athletic performance is reported to begin at ages 12–13 years, reaching an adult plateau in late adolescence [36]. These findings parallel the results of the current study, where children aged 12 and older produced higher power. Sex differences become more pronounced between the ages of 14 and 15, likely reflecting the growth spurt in muscle mass during adolescence.

### Sex and sprinting performance

In examining sex differences in sprinting performance and fatigue in children aged 9–14, it is essential to understand the underlying physiological differences between boys and girls during this developmental stage. These differences primarily stem from biological factors, which significantly impact athletic performance [38]. Prior to puberty, however, there are no significant sex-based differences in testosterone levels, and performance is typically more comparable between boys and girls [39, 41]. Yet boys outperform girls in competitive sprinting and shuttle run tests even before puberty. Boys have faster growth velocities and accumulate less fat mass than girls during childhood and these sex differences in body composition may confer an athletic advantage among boys compared with girls before ages associated with puberty and adolescence [20, 36, 37, 40]. Atkinson et al. [21] found that male youth outperformed female youth across various track running events (100-, 200-, 400-, and 800-m), with the performance gap widening as age increased. This is consistent with other studies showing that boys outperform girls in strength, power, endurance, and speed/agility tests, with these differences becoming more pronounced around age 11 [11, 42, 43, 44].

During puberty hormonal influences significantly diverge athletic performance between males and females. The mechanisms behind these differences in performance are complex, with muscle mass, cardiovascular capacity all playing a role. Boys generally have more muscle mass and greater cardiovascular capacity (larger heart size, larger stroke volume, and higher blood hemoglobin concentration) than girls, which allows them to perform better in tasks requiring strength and endurance, such as sprinting [39]. In contrast, girls may experience physical changes (e.g., increased fat deposition [40] which may limit their sprinting performance.

### Heart rate

Heart rate was a key marker of exertion, increasing significantly over the six runs (very large effect size). However, no sex or age differences were found in either peak or mean heart rate, which is in contrast to some previous studies [45] who found that heart rates in trained girls aged 12 to 15 were consistently higher than those of boys at every running speed during graded exercise. The differences in heart rate may be influenced by factors like age, sex and physical activity level, as noted by Sharma et al. [46], who identified these as primary determinants of heart rate variability in adolescents aged 12–17. The lack of difference may be due to the specific nature of the sprinting protocol and the fitness levels of participants. For instance, differences in heart rate responses may vary based on the intensity or type of exercise, as well as the participant’s training status. Future research should further explore these variables.

### Fatigue

Although the literature on sex differences in performance is extensive, less is known about the key factor of fatigue. Fatigue, defined as the gradual deterioration of motor performance due to prolonged exertion [47, 48], can vary between males and females and is influenced by multiple factors, including muscle fiber composition, metabolic rate, and hormonal influences. Although the current study confirmed that girls ran slower than boys, their times were similar in the first three runs of the test, with increased running times observed in the last three. It has been stated that men and women have different metabolic and hormonal responses to maximal exercise [49, 50], and that males may experience less fatigue during high-intensity tasks due to their higher muscle mass and greater ability to generate force [51, 52]. This could partly explain why boys maintain better performance during repeated sprints and experience slower declines due to fatigue.

The lack of differences in maximum and estimated heart rates suggests comparable effort levels between boys and girls, although psychological factors, such as competitiveness, may also play a role. Furthermore, the lack of sex differences in power challenges the assumption that girls “give up” earlier than boys. However, the literature on fatigue in young girls is scarce. Research on muscle fatigue in adults suggests that women generally have longer endurance times than men, especially at low-to-moderate forces, even when exercise intensity is matched. This is attributed to greater oxygen availability during exercise, which allows females to sustain higher relative intensities than males [53].

In conclusion, sex differences in running times, power, and fatigue in this age group can largely be attributed to physiological disparities between boys and girls, which become more evident around puberty. Males experience a dramatic increase in testosterone during puberty [36, 39, 54],, which results in greater muscle mass, stronger bones, larger hearts and lungs, and enhanced oxygen-carrying capacity due to higher hemoglobin levels. These physiological changes contribute to superior athletic performance in males, particularly in terms of running speed and fatigability. Importantly, anaerobic performance is only one aspect of physical fitness development, and as youth progress through puberty, running skills become incorporated into a variety of more sport-specific skills [55]. While the focus is still on skill training, functional strength, and speed, aerobic training become increasingly important around age 12 in boys and age 11 in girls. These aspects should be considered in physical assessments of this age group.

## Strengths and limitations

This study contributes valuable insights into age- and sex-related differences in anaerobic performance, using the CRISP test to evaluate fatigue, power, and running speed. However, it has limitations. The study only measured performance fatigability (i.e., decay a running time), which does not capture perceived fatigue or exhaustion. Including assessments of perceived fatigue would add depth to our understanding of how children experience fatigue during high-intensity exercise. Further, assessing lean body mass would help clarify the relationship between muscle mass and sprint performance, particularly in light of sex-based differences during puberty. Additionally, larger sample sizes per age group and varying training levels would provide more robust data on sex differences in fatigue.

## Conclusion

This study confirmed that children experience significant fatigue during the six runs of the CRISP test, with heart rate increasing by 30% across the test. Children reached about 66% of their estimated maximum heart rate during the approximately 6-second runs. Although there were no sex or age differences in heart rate responses, significant differences were observed in running performance, with older children producing more power and boys generally running faster than girls. The CRISP test effectively distinguishes performance characteristics by age and sex, providing a useful tool for tracking the development of sprinting and anaerobic capacity in children. This test may also help guide physical education programs, where older children tend to exhibit higher power but performance differences between boys and girls remain small until puberty. Consequently, both boys and girls can follow similar PE classes during the prepubertal years but when the performance gap widens, girls should be provided with an educational environment that secures equal opportunities for physical fitness development.

## Data Availability

All data needed for the analysis have been entered in the tables. Datasets used and/or analyzed during the current study are available from the corresponding author on reasonable request.

## Abbreviations

CRISP: Children’s repetitive and intermittent sprinting performance
RAST: Run-based anaerobic sprint test
PP: Peak power
MP: Mean power
BMI: Body mass index
HR: Heart rate
EMHR: Estimated maximum heart rate
